# In Vitro Sensitivity of Leukemia Cells to Propranolol

**DOI:** 10.4021/jocmr2009.06.1244

**Published:** 2009-07-03

**Authors:** Fatemeh Hajighasemi, Abbas Mirshafiey

**Affiliations:** a Department of Immunology, Faculty of Medicine, Shahed University, Tehran, Iran.; b Department of Pathobiology, School of Public Health, Tehran University of Medical Sciences, Tehran, Iran.

## Abstract

**Background:**

Propranolol, as a beta-adrenergic blocker is used for treatment of a large number of cardiovascular diseases such as hypertension and arrhythmias. The inhibitory effects of propranolol on tumor cells growth and also its cytotoxicity on cancerous cells have been revealed by several studies. In this study the sensitivity of a number of human leukemic cell lines to propranolol was evaluated in vitro.

**Methods:**

Two human leukemic T cells (Molt-4 and Jurkat) and a monocyte (U937) cell line were used in this study. The cells were cultured in complete RPMI medium and then incubated with different concentrations of propranolol (0.0004 -0.4 mM) in the presence or absence of phytoheamagglutinin (20 μg/ml) for 12, 24 and 48 hours. The cytotoxic effect of the drug was then assessed by trypan blue dye exclusion and also 3-(4,5-dimethyl thiazol-2,5-diphenyltetrazoliumbromide) (MTT) reduction methods.

**Results:**

Propranolol induced a significant dose dependent cytotoxic effect at ≥ 0.2 mM concentration on all three human cell lines (Molt-4, Jurkat and U937) used in this study, after 12 hours incubation onwards, compared to untreated control cells.

**Conclusions:**

Our results demonstrated that leukemic cell lines used in this study were sensitive to propranolol at ≥ 0.2 mM concentration of the drug. These results suggest that propranolol may have potential implication in chemoprevention of lymphoproliferative disorders along with its chronic long-term usage in cardiac problems.

**Keywords:**

Propranolol; Leukemia; Cell lines; Sensitivity

## Introduction

Propranolol, as a non selective beta-adrenergic blocker, has been extensively used for treatment of many cardiovascular problems such as hypertension and arrhythmias [1, 2]. It has been recently suggested that beta-blockers decrease tumor progression through suppression of cancerous cells proliferation, inhibition of growth factor production and apoptosis induction of tumor cells [3]. The inhibitory effect of propranolol on phospholipase D pathway, through blocker mechanism, resulting in decreased phosphatidic acid (PA) production has been shown [4, 5]. PA is a necessary component for phospholipids biosynthesis and tumor cell growth [5]. Propranolol inhibitory effects on a tobacco-induced pulmonary adenocarcinoma development [6], uterine leiomyoma induction [7], human lung adenocarcinoma cell line proliferation [8], surgery increased lung tumor retention [3] and TNF- alpha induced proliferation of rat C6 glioma cells [9] have been reported. In addition, the cytotoxic effects of propranolol on rat and human lung cells [10], human skin keratinocytes, fibroblasts, corneal and retinal epithelial cell lines [11] and also tumor cells have been revealed [5]. Moreover increase in splenocyte apoptosis rate and decrease of proliferative capacity of splenocytes after administration of propranolol in septic mice has been demonstrated [12, 13]. Furthermore anti-inflammatory effects of chronic exposure to beta-blockers have been reported [14, 15]. Besides the attenuating effect of propranolol on proinflammatory cytokines such as IL-1 beta mRNA expression [16] and TNF-alpha serum level in migraine patients has been shown [17].

Based on the anti tumor and anti-inflammatory properties of propranolol, the present study was conducted to examine the sensitivities of three human leukemic cell lines to propranolol in vitro.

## Materials and Methods

### Reagents

RPMI-1640 medium, penicillin, streptomycin, phytoheamagglutinin (PHA), and trypan blue (TB) were purchased from Sigma (USA). Fetal calf serum (FCS) was obtained from Gibco (USA) and MTT (3-(4,5-dimethyl thiazol-2,5-diphenyltetrazoliumbromide)) kit was purchased from invitrogen (USA). Propranolol was a kind gift from HAKIM Pvt. Co. Ltd (Tehran, Iran). Microtiter plates, flasks and tubes were purchased from Nunc (Falcon, USA).

### Preparation of propranolol

Propranolol was dissolved in RPMI-1640 medium and stored at -20^o^C until use. Drug was diluted in culture medium to prepare appropriate concentrations before use.

### Cell lines

Human leukemic T cells [Molt-4 (NCBI C149) and Jurkat (NCBI C121)] and monocyte [U937 (NCBI C130)], were obtained from NCBI (National Cell Bank of Iran, Pasteur Inst. of Iran, Tehran). The cells were maintained in RPMI-1640 medium supplemented with 10% FCS in 5% CO_2_ at 37^o^C.

### Cell culture and treatment

Human leukemic cells were cultured in RPMI-1640 medium supplemented with 10% FCS, penicillin (100 IU/ml) and streptomycin (100 g/ml) at 37^o^C in 5% CO_2_. The cells were seeded at a density of 3 x 10^4^ cell/well and then incubated with different concentrations of propranolol (0.0004 - 0.4 mM) in the presence or absence of PHA (20 μg/ml) for 12, 24 and 48 hours. All experiments were done in triplicate.

### Cell proliferation assay

To evaluate the effect of different concentrations of drug on viability of leukemic cell lines, we used trypan blue dye exclusion (TB test) [18] and MTT assay [19].

### Trypan blue dye exclusion test

Principle of trypan blue dye exclusion test is exclusion of dye by viable cells and taking it up by dead cells. Viability is evaluated by direct counting of viable and dead cells. Percentage of the number of viable cells to the total number of cells is considered as viability percentage.

### MTT assay

In MTT test the conversion of yellow water soluble MTT to a blue-insoluble formazon was assessed according to the method developed by Mosmann [19]. At the end of incubation time, the medium was replaced with 100 μl of fresh medium. The amount of 10 μl of MTT solution (5 mg/ml in PBS) was then added to each well and incubated at 37^o^C for 4 hours. Subsequently 100 μl of the SDS-HCl solution (100 mg SDS was dissolved in 1 ml HCl) was added to each well and incubated at 37^o^C for 4 hours. So the insoluble formazon derivative was dissolved and absorbance at 570 nm was measured using a microplate reader (Awarness Technology INC). The results were expressed as cell numbers per control.

### Statistical analysis

Effect of the drug on each cell line was performed in three independent experiments (n = 3) and the results were expressed as mean ± SD. Statistical comparisons between groups were made by analysis of variance (ANOVA). P < 0.05 was considered significant. Test of multiple comparison of Tukey was applied (5%) for statistically significant differences. For statistical analysis and graph making the software SPSS 16.0 and Excel 2003 were used respectively.

## Results

**Figure 1:**
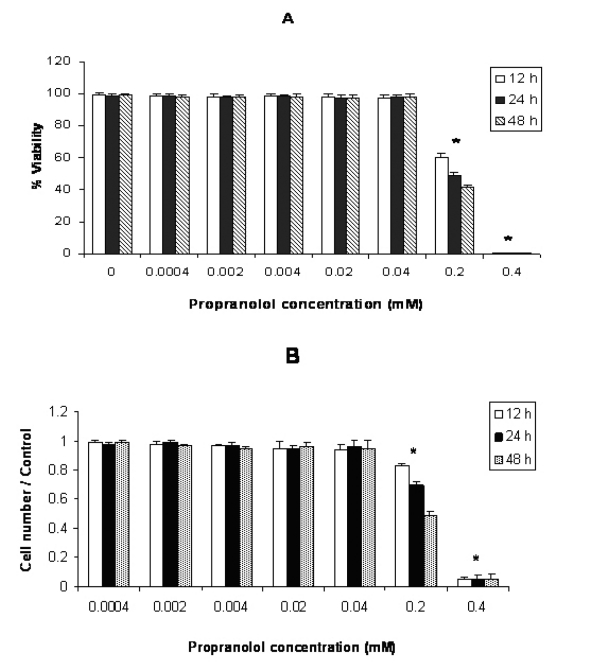
Effect of propranolol on proliferative responses of human leukemic Molt-4 T-cell line. The Molt-4 cells were treated with different concentrations of propranolol (0.0004 - 0.4 mM) for 12, 24 and 48 hours. The results are presented as % of viability demonstrated by trypan blue dye exclusion (TB) test (A) and cell number/control demonstrated by MTT assay (B). Data are mean ± SD of triplicate cultures. n = 3; P < 0.05 was considered significant.

**Figure 2:**
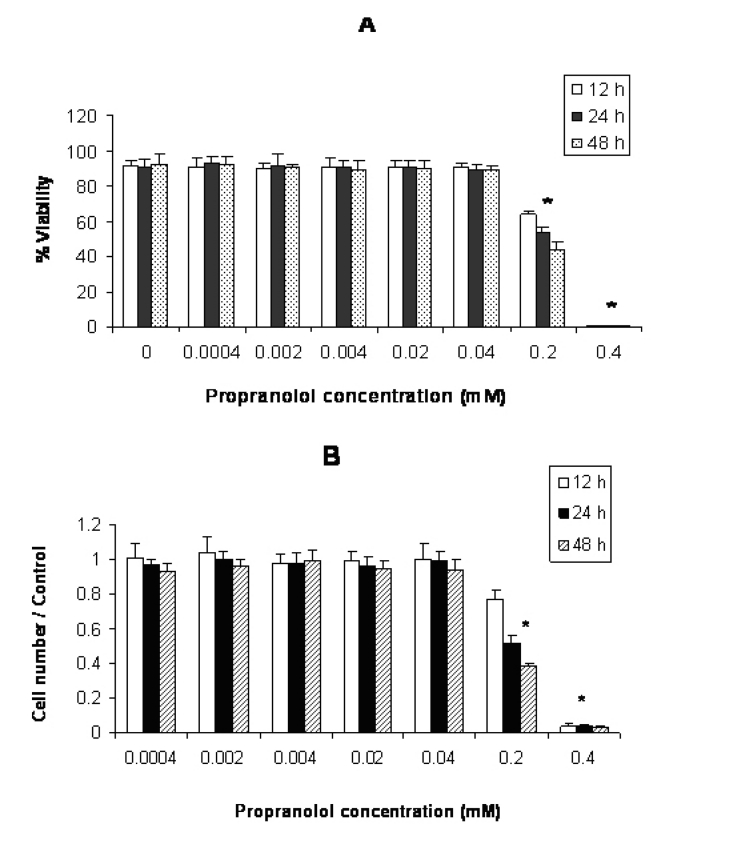
Effect of propranolol on proliferative responses of human leukemic Jurkat T-cell line. The Jurkat cells were treated with different concentrations of propranolol (0.0004 - 0.4 mM) for 12, 24 and 48 hours. The results are presented as % of viability demonstrated by trypan blue dye exclusion (TB) test (A) and cell number/control demonstrated by MTT assay (B). Data are mean ± SD of triplicate cultures. n = 3; P < 0.05 was considered significant.

**Figure 3:**
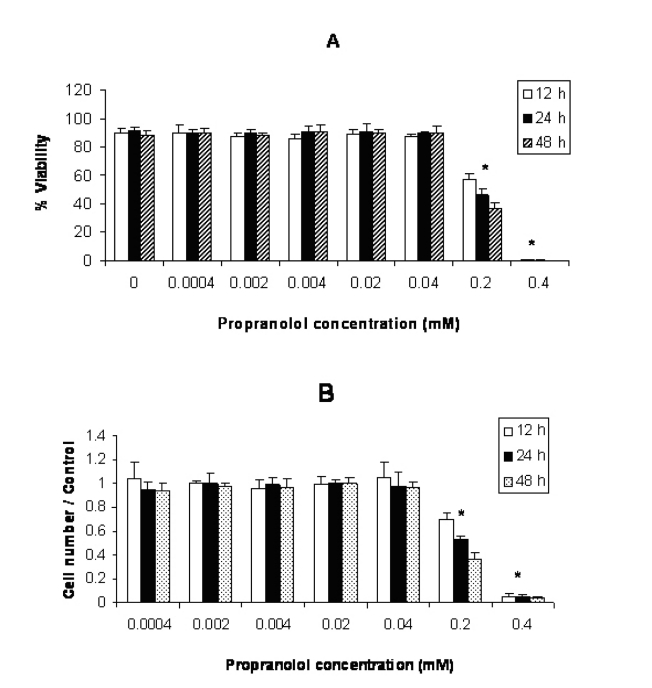
Effect of propranolol on proliferative responses of human leukemic U937 cell line. The U937 cells were treated with different concentrations of propranolol (0.0004 - 0.4 mM) for 12, 24 and 48 hours. The results are presented as % of viability demonstrated by trypan blue dye exclusion (TB) test (A) and cell number/control demonstrated by MTT assay (B). Data are mean ± SD of triplicate cultures. n = 3; P < 0.05 was considered significant.

Cytotoxic effect of propranolol on human leukemic cell lines in different concentrations and time intervals are shown in figures 1, 2 and 3. In each figure, A and B represent the results of trypan blue dye exclusion and MTT assays respectively. Propranolol significantly decreased proliferative responses of human leukemic cell lines in both staining methods in all time intervals dose-dependently (P < 0.05) (Fig. 1-3). The results depicted in Figure 1 (A and B) showed that propranolol significantly decreased the proliferation of Molt-4 cells at ≥ 0.2 mM concentration after 12 hours incubation compared with untreated control cells (P < 0.05).

According to Figure 2(A and B), propranolol significantly inhibited proliferation of Jurkat cells at ≥ 0.2 mM propranolol concentration after 12 hours incubation compared with untreated control cells (P < 0.05 ).

The results illustrated in Figure 3(A and B) showed that incubation of U937 cells with propranolol led to significant inhibition of cell proliferation at ≥ 0.2 mM concentration (P < 0.05 ) after 12 hours incubation compared with untreated control cells.

Propranolol cytotoxicity at 0.2 mM concentration was significantly increased with time in this order: 48h > 24 h > 12 h in all cell lines (Fig. 1-3. Similar results were obtained after stimulation of propranolol treated cells with PHA (data not shown).

## Discussion

This study was carried out to determine the effects of propranolol on proliferative response of human leukemic T and monocyte cell lines. Our results demonstrated that all three cell lines used in this study were sensitive to propranolol after 12 hours incubation time onwards at ≥ 0.2 mM drug concentration (Fig. 1-3). So sensitivity of different leukemic cell lines was similar to each other indicating that toxic mechanism is not cell specific. Similarly Cheong et al [11] reported just little differences in toxicity of a number of beta-blockers including propranolol on human epithelial cell lines, epidermal keratinocytes and dermal fibroblasts for each drug. Conversly, Kastelova et al [10] showed propranolol cytotoxicity at different (0.001- 1 mM) concentrations in rat and human alveolar macrophages, rat type II pneumocytes and human lung adenocarcinoma cell line A549 [12]. The different sensitivities of the cells in the study of Kastelova et al to propranolol may be in part due to using different cell types (normal and cancerous) with different origin (rat and human).

Our data along with Kastelova et al [10] revealed that the propranolol could show a significant cytotoxicity at relatively high concentration of this drug. In Kastelova et al study, longer incubation time with propranolol increased its cytotoxicity in ≥ 0.5 mM concentration of the drug [10]. In our study, although propranolol cytotoxicity at 0.4 mM concentration was significantly higher than 0.2 mM, a significant increase in cytotoxicity with time (48 h > 24 h > 12 h) only was observed at 0.2 mM concentration of the drug. This discrepancy between Kastelova et al and our study may be due to that Kastelova et al assessed propranolol cytotoxicity after 3 and 20 hours but in our study time intervals were 12, 24 and 48h. Thus Kastelova et al started propranolol cytotoxicity evaluation after 3h incubation which was very earlier than our first time interval assessment (12h). In our study, more than 99% of the cells died at 0.4 mM concentration of the drug after 12h incubation and there was no significant difference in propranolol cytotoxicity between different incubation times at 0.4 mM dose.

However in our study, longer incubation time with propranolol, increased its cytotoxic effect at 0.2 mM concentration. Prolongation of incubation time may increase propranolol concentration inside the cell. Propranolol as a cationic amphiphilic drug can accumulate in particular sub cellular organs resulting in greatly higher local concentrations from that in the medium [20]. Accumulation of propranolol in lysosomes and non specific binding of it to animal microsomes have been reported [21]. Furthermore repeated administration of propranolol caused cytotoxicity at lower doses compared to single treatment of hepatoma cell line HBG BC2 [22]. So continual uses of propranolol in patients, possibly, make it cytotoxic at lower concentrations in vivo compared to in vitro usage of this drug.  In addition chronic administration of propranolol in ulcer patients resulted in a minor inhibitory effect on proliferation of PHA-stimulated lymphocytes [23]. Also in rat model, continual administration of propranolol strongly inhibits hepatic metabolism and has different effects on rat lung type II pnemocytes and alveolar macrophage xenobiotic metabolizing enzyme activities [24]. Covalent binding of metabolic intermediates of propranolol to some liver isoenzymes has also been reported [25].

Our results along with other investigators findings suggest that propranolol, in addition to its primary action on beta-adrenergic receptors, may modulate cellular functions at higher concentrations. We also evaluated the effect of propranolol on leukemic cells in the presence of PHA. We did not find any difference in propranolol cytotoxicity in the presence or absence of PHA )data not shown). This suggests that PHA dependent proliferative mechanisms are also sensitive to propranolol.

While the anti-tumoral and anti-inflammatory properties of propranolol on a variety of carcinomas have been shown by some investigators [6-9, 15-17] and its antiproliferative effect on some normal cells was observed in its continuing use [25], the cytotoxic effects of this drug on normal cells, at its effective anti tumoral concentration has not been reported yet. Accordingly it could be noteworthy to assess its cytotoxic effect on normal cells as well as tumor cells in a long time period in vivo to determine the most effective anti- tumoral dose of the drug with regard to lowest cytotoxic effects on normal body components in a special period of time.

As a whole our results suggest that propranolol could be a possible useful agent for chemoprevention of leukemic disorders along with its chronic long-term usage in cardiac problems.

## References

[1] Priviero FB, Teixeira CE, Claudino MA, De Nucci G, Zanesco A, Antunes E (2007). Vascular effects of long-term propranolol administration after chronic nitric oxide blockade. Eur J Pharmacol.

[2] Degoute CS (2007). Controlled hypotension: a guide to drug choice. Drugs.

[3] Benish M, Bartal I, Goldfarb Y, Levi B, Avraham R, Raz A, Ben-Eliyahu S (2008). Perioperative use of beta-blockers and COX-2 inhibitors may improve immune competence and reduce the risk of tumor metastasis. Ann Surg Oncol.

[4] Bhat RS, Bhaskaran M, Mongia A, Hitosugi N, Singhal PC (2004). Morphine-induced macrophage apoptosis: oxidative stress and strategies for modulation. J Leukoc Biol.

[5] Finney RE, Nudelman E, White T, Bursten S, Klein P, Leer LL, Wang N (2000). Pharmacological inhibition of phosphatidylcholine biosynthesis is associated with induction of phosphatidylinositol accumulation and cytolysis of neoplastic cell lines. Cancer Res.

[6] Schuller HM, Porter B, Riechert A (2000). Beta-adrenergic modulation of NNK-induced lung carcinogenesis in hamsters. J Cancer Res Clin Oncol.

[7] Gibson JP, Sells DM, Cheng HC, Yuh L (1987). Induction of uterine leiomyomas in mice by medroxalol and prevention by propranolol. Toxicol Pathol.

[8] Schuller HM, Cole B (1989). Regulation of cell proliferation by beta-adrenergic receptors in a human lung adenocarcinoma cell line. Carcinogenesis.

[9] Lung HL, Shan SW, Tsang D, Leung KN (2005). Tumor necrosis factor-alpha mediates the proliferation of rat C6 glioma cells via beta-adrenergic receptors. J Neuroimmunol.

[10] Kastelova A, Dimova S, Nemery B (2003). Propranolol cytotoxicity in rat and human lung in vitro. Methods Find Exp Clin Pharmacol.

[11] Cheong HI, Johnson J, Cormier M, Hosseini K (2008). In vitro cytotoxicity of eight beta-blockers in human corneal epithelial and retinal pigment epithelial cell lines: comparison with epidermal keratinocytes and dermal fibroblasts. Toxicol In Vitro.

[12] Schmitz D, Wilsenack K, Lendemanns S, Schedlowski M, Oberbeck R (2007). beta-Adrenergic blockade during systemic inflammation: impact on cellular immune functions and survival in a murine model of sepsis. Resuscitation.

[13] Oberbeck R, Schmitz D, Wilsenack K, Schuler M, Pehle B, Schedlowski M, Exton MS (2004). Adrenergic modulation of survival and cellular immune functions during polymicrobial sepsis. Neuroimmunomodulation.

[14] Sood AK, Bhatty R, Kamat AA, Landen CN, Han L, Thaker PH, Li Y (2006). Stress hormone-mediated invasion of ovarian cancer cells. Clin Cancer Res.

[15] Nguyen LP, Omoluabi O, Parra S, Frieske JM, Clement C, Ammar-Aouchiche Z, Ho SB (2008). Chronic exposure to beta-blockers attenuates inflammation and mucin content in a murine asthma model. Am J Respir Cell Mol Biol.

[16] Deten A, Volz HC, Briest W, Zimmer HG (2002). Cardiac cytokine expression is upregulated in the acute phase after myocardial infarction. Experimental studies in rats. Cardiovasc Res.

[17] Covelli V, Passeri ME, Leogrande D, Jirillo E, Amati L (2005). Drug targets in stress-related disorders. Curr Med Chem.

[18] Moldeus P, Hogberg J, Orrenius S (1978). Isolation and use of liver cells. Methods Enzymol.

[19] Mosmann T (1983). Rapid colorimetric assay for cellular growth and survival: application to proliferation and cytotoxicity assays. J Immunol Methods.

[20] Schneck DW, Pritchard JF, Hayes AH, Jr (1977). Studies on the uptake and binding of propranolol by rat tissues. J Pharmacol Exp Ther.

[21] Cramb G (1986). Selective lysosomal uptake and accumulation of the beta-adrenergic antagonist propranolol in cultured and isolated cell systems. Biochem Pharmacol.

[22] Fabre N, Arrivet E, Trancard J, Bichet N, Roome NO, Prenez A, Vericat JA (2003). A new hepatoma cell line for toxicity testing at repeated doses. Cell Biol Toxicol.

[23] Malec PH, Zeman K, Markiewicz K, Tchorzewski H (1990). Chronic beta-adrenergic antagonist treatment affects human T lymphocyte responsiveness "in vitro". Allergol Immunopathol.

[24] Kastelova A, Dimova S, Nemery B (2003). Effects of propranolol on xenobiotic enzyme activities in rat type II pneumocytes and alveolar macrophages in vivo. Methods Find Exp Clin Pharmacol.

[25] Narimatsu S, Watanabe T, Masubuchi Y, Horie T, Kumagai Y, Cho AK, Imaoka S (1995). Characterization of a chemically reactive propranolol metabolite that binds to microsomal proteins of rat liver. Chem Res Toxicol.

